# Phenome-wide association study for *CYP2A6* alleles: rs113288603 is associated with hearing loss symptoms in elderly smokers

**DOI:** 10.1038/s41598-017-01098-4

**Published:** 2017-04-21

**Authors:** Renato Polimanti, Kevin P. Jensen, Joel Gelernter

**Affiliations:** 1 Department of Psychiatry, Yale University School of Medicine and VA CT Healthcare Center, West Haven, CT United States; 2 Departments of Genetics and Neuroscience, Yale University School of Medicine, New Haven, CT United States

## Abstract

To identify novel phenotypic associations related to *Cytochrome P450 Family 2 Subfamily A Member 6* (*CYP2A6*), we investigated the human phenome in a total of 11,271 individuals. Initially, we conducted a phenome-wide association study in 3,401 nicotine-exposed elderly subjects considering 358 phenotypic traits. We identified a significant association between *CYP2A6* rs113288603 and hearing loss symptoms (p = 5.75 × 10^−5^). No association was observed in a sample of 3,245 nicotine-unexposed individuals from the same discovery cohort, consistent with the conclusion that the finding is related to *CYP2A6* involvement in nicotine metabolism. Consistent results were obtained (p < 0.1) in an independent sample of 2,077 nicotine-exposed elderly subjects, and similarly, no significance was observed in the nicotine-unexposed sample (n = 2,548) of the replication cohort. Additional supporting evidence for this association was provided by gene expression data: rs113288603 is associated with increased *CYP2A6* expression in cerebellar hemispheres (p = 7.8 × 10^−4^). There is a well-known correlation between smoking and age-related hearing loss. Cigarette smoking is associated with structural changes in the brain and *CYP2A6* mediates these changes. In this context, the regulatory role of rs113288603 in cerebellum appears to be consistent with the known involvement of this brain region in auditory function.

## Introduction

Cigarette smoking causes nearly one in five deaths in the United States and is recognized as one of the most dangerous risk factors for numerous cancers and chronic diseases (information from Center for Disease Control and Prevention available at https://www.cdc.gov/tobacco/data_statistics/fact_sheets/health_effects/effects_cig_smoking/). Although the harmful consequences of smoking behaviors are widely recognized, many of the mechanisms of the biochemistry by which it affects human health are still unknown. Indeed, beyond the several smoking-disease correlations currently recognized^1, 2^, there are likely many additional associations to be revealed, especially as ever-larger datasets containing various kinds of medical data become available to researchers. Genetic investigations, such as phenome-wide association studies (PheWAS, i.e., association analysis of known functional alleles with respect to a large number of phenotypes) of known risk alleles can verify association hypotheses and identify novel medically-relevant associations^3, 4^. Regarding tobacco smoking, several loci have been identified and confirmed by multiple independent studies. The best replicated so far are risk alleles located in the *CHRNA3–CHRNA5–CHRNB4* gene cluster that were associated with smoking behaviors (e.g., cigarettes per day) and smoking-associated diseases (e.g., lung cancer)^5–7^. Beyond this locus, another relevant gene is *CYP2A6* (*Cytochrome P450 Family 2 Subfamily A Member 6*). Its protein product is involved in the nicotine metabolic pathway and its functional alleles have large effects on an index of CYP2A6 activity, the nicotine metabolite ratio (NMR)^8–10^, explaining a strikingly large fraction of the variance (up to 31%)^8^. It also has numerous other metabolic functions^11^. In our previous PheWAS for *CHRNA3–CHRNA5–CHRNB4* risk alleles, we confirmed the association for smoking behaviors and known smoking consequences (e.g., lung cancer and asthma) and identified potential phenotypic associations related to human behaviors and lipid metabolism^12^.

Due to the relevance of *CYP2A6* in nicotine metabolism and the growing literature regarding the potential dangerous consequences of exposure to nicotine from tobacco cigarettes and newer generation tobacco products (e.g., electronic cigarettes)^13–15^, we conducted a 360-trait PheWAS for nine putative functional *CYP2A6* alleles in nicotine-exposed subjects from the Women’s Health Initiative cohort (WHI)^16^. Our main finding was related to the association of *CYP2A6* rs113288603 with hearing loss; there is a known correlation between smoking and age-related hearing loss^17, 18^. The Women’s Health Initiative cohort includes women in menopause (average age: 61 yrs) and hearing loss in this cohort is considered to be attributable largely to aging processes. This result was replicated in an independent cohort (average age = 69 yrs) from the Long Life Family Study (LLFS)^19^. We further observed that rs113288603 is associated with *CYP2A6* expression in cerebellar hemispheres, which are involved in auditory function^20, 21^. Our findings confirm the role of tobacco smoking in age-related hearing loss and suggest that *CYP2A6* mediates this association (at least in part) by moderating the long-term effects of nicotine or an ototoxic metabolite in the brain to various degrees depending on the individual.

## Methods

### Study Populations

The datasets used for the analyses were obtained, after authorized access, from the National Center for Biotechnology Information (NCBI) database of Genotypes and Phenotypes (dbGaP; available at http://www.ncbi.nlm.nih.gov/gap) through dbGaP accession numbers: phs000200.v9.p3 for the WHI Clinical Trial and Observational Study^16^; and phs000397.v1.p1 for National Institute on Aging (NIA) Long Life Family Study (LLFS)^19^. All dbGaP dataset versions used in the current study are past their embargo periods. The Yale University Institutional Review Board approved the secondary analysis of these dbGaP datasets. WHI and LLFS studies were conducted in accordance with the relevant guidelines and regulations and their participants signed the informed consent to permit secondary analyses of their data.

A detailed description of the phenotypes and the procedures used for data extraction and quality control of the WHI dataset is available in our previous PheWAS^12^. In summary, the initial PheWAS conducted on the WHI cohort that included 360 traits (Supplemental Table 1) related to 12 main categories: Anthropometric traits, Cancer, Cardiovascular Health, Dietary Habits, Drinking Behaviors, Gastrointestinal Health, General Health, Physical Activity, Psychological Traits, Reproductive Health, Smoking Behaviors, and Socioeconomic Status. As further clarified in the *Genotype Data* section, we considered the WHI SHARe dataset and focused the analysis on individuals of African descent because they represent the large majority of this cohort. The LLFS cohort, which includes individuals of European descent, was used to address replication; we used the same procedures for phenotype extraction and quality control as applied to the WHI cohort. We note in particular, the “hearing loss” item (because this phenotype generated the most relevant result in the PheWAS conducted) was similarly assessed in both cohorts: WHI – “*Hearing loss: Symptom was severe; Symptom was moderate; Symptom was mild; Symptom did not occur*”; LLFS – “*Respondent able to hear: No; Yes with great difficulty; Yes with little difficulty; Yes without any difficulty*”. Both cohorts were stratified for nicotine exposure (more than 100 cigarettes lifetime) and the subsequent analyses were conducted accordingly (exposed *vs*. unexposed). Because large GWAS cohorts of elderly subjects assessed for hearing loss are limited, we investigated discovery and replication cohorts with different ancestry backgrounds. Previous trans-population studies of *CYP2A6* have been conducted successfully^9, 10^.

### Genotype Data

For our PheWAS, we investigated multiple alleles mapped to the *CYP2A6* locus based on previous evidence of nicotine-related functional effects. These variants were selected from the significant results of a recent genome-wide association study (GWAS) of NMR^8^ and from known functional *CYP2A6* alleles from the Human Cytochrome P450 (CYP) Allele Nomenclature (available at http://www.cypalleles.ki.se/cyp2a6.htm). For the WHI cohort, we considered the WHI SHARe dataset (phs000386.v5.p3) because it includes the genome-wide data needed to impute *CYP2A6* alleles. A detailed description of the procedures used for genotyping, genotype quality control, principal component analysis, and imputation of the WHI dataset is found in our previous PheWAS^12^. Briefly, we conducted a principal component analysis using Plink 1.9^22^ and genome-wide datasets pruned for linkage disequilibrium (r^2^ > 80%). Genotype imputation was performed using SHAPEIT^23^ for pre-phasing, IMPUTE2^24^ for imputation, and the 1000 Genomes Project Phase 3^25^ as the reference panel. After imputation, we obtained good-quality genotype dosage information for nine *CYP2A6* variants (info score > 0.5; Supplemental Table 2), including three independent genome-wide significant SNPs identified by the NMR GWAS^8^ and six functional *CYP2A6* alleles from the Human Cytochrome P450 Allele Nomenclature (inclusion criteria for the definition of functional CYP alleles are available at http://www.cypalleles.ki.se/criteria.htm). The final WHI sample size was 3,401 nicotine-exposed and 3,245 nicotine-unexposed individuals. For the LLFS cohort, genotyping was conducted using the Illumina HumanOmni2.5. We applied the same criteria for quality control and imputation as previously applied to the WHI cohort. Because LLFS cohorts includes related individuals, we used the PC-AiR (Principal Components Analysis in Related Samples) algorithm in the R Package GENESIS (available at https://bioconductor.org/packages/release/bioc/html/GENESIS.html) for the principal component analysis. After quality control, the final LLFS sample size was 2,077 nicotine-exposed and 2,548 nicotine-unexposed individuals – substantially smaller than the discovery sample.

### Statistical Analysis

The discovery PheWAS was conducted in the WHI nicotine-exposed sample using Plink 1.9^22^. Logistic and linear regression analyses were used to calculate the association between genetic variants and phenotypes (binary and quantitative, respectively). The regression models included as covariates age, age-squared, and the first 10 principal components to control for differences in ancestry. Since PheWAS are not discovery studies (they are follow-up investigations useful to delineate the role of previously identified loci in the human phenome), they can be corrected with less stringent multiple testing criteria than the Bonferroni correction for the number of independent tests^3, 26, 27^. We adjusted our p values using a locus-wise Bonferroni correction (which corresponds to correction for the number of phenotypes studied) that was recently proposed by Simonti and colleagues^27^. Accordingly, we calculated that the phenome-wide significance threshold to keep the type I error rate at 5% is p = 1.40 × 10^−4^. Since the LLFS cohort is ~40% smaller than WHI cohort (i.e., the association analysis in the replication cohort had Lower statistical power than that conducted on the discovery cohort), we considered p = 0.1 as indicative of successful replication, while bearing in mind that this “trend-level” requirement is a necessary compromise and true replication will require additional subject samples. To verify whether the association observed were due to the role of CYP2A6 in nicotine metabolism, we conducted an interaction test, analyzing the difference between regression coefficients in nicotine-exposed vs. nicotine-unexposed subjects. Because the LLFS cohort includes closely related subjects, we performed the association and interaction analyses using the R package GWAF^28^ to fit a generalized estimating equations (GEE) model to adjust for correlations among related individuals. Genotype-Tissue Expression (GTEx) Version 6 data (available at http://www.gtexportal.org/) were used to analyze the effect of alleles investigated on *CYP2A6* expression across 36 human tissues^29^. A detailed description of the GTEx methods (i.e., preprocessing, expression quantification, and association analysis) used is available at http://www.gtexportal.org/static/doc/analysis/Portal_Analysis_Methods_v6_08182016.pdf. Briefly, the association between genetic variant and gene expression was conducted using a linear regression analysis considering the covariates (i.e., top-3 ancestry principal components, genotyping array platform, PEER factors, and sex) applied to gene-cis-SNP pairs using Matrix eQTL^30^ and assuming an additive model.

## Results

**Figure 1:**
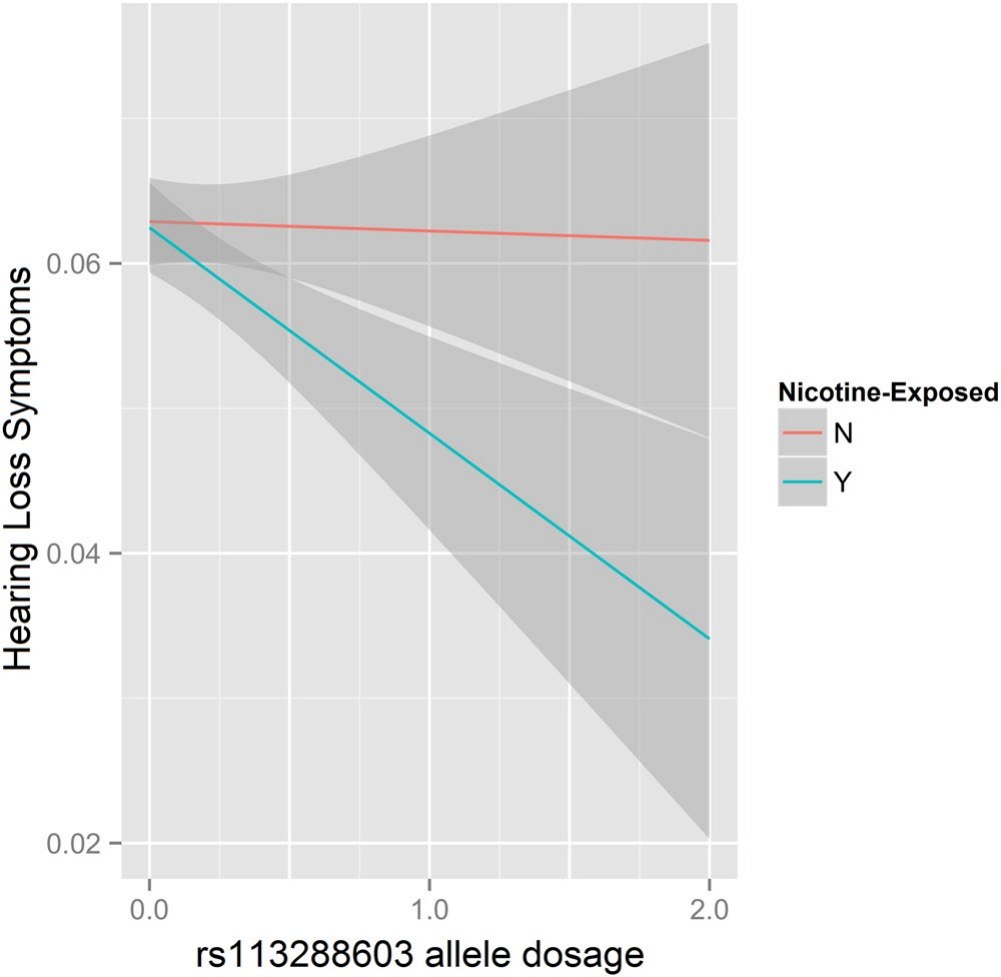
Differences in the association between *CYP2A6* rs113288603*T and hearing loss symptoms in nicotine-exposed and nicotine-unexposed subjects from the WHI cohort.

In the discovery PheWAS in the WHI nicotine-exposed sample, we observed a significant association between *CYP2A6* rs113288603 and hearing loss. The minor allele T was associated with reduced symptoms, i.e., it was protective (p = 5.75 × 10^−5^; Table 1). To test whether the hearing-loss association was dependent on nicotine exposure, we analyzed the WHI nicotine-unexposed sample. No association was observed between *CYP2A6* rs113288603 and hearing loss symptoms (p = 0.871) among unexposed subjects, and a significant difference was observed between nicotine-exposed and nicotine-unexposed results (p_Interaction_ = 8.67 × 10^−3^; Fig. 1). To confirm the finding from the discovery PheWAS, we conducted an additional analysis in the LLFS cohort, where we observed a replication of the negative association between *CYP2A6* rs113288603 and hearing loss symptoms in nicotine-exposed subjects (p = 0.098), and as with the discovery sample, no association was observed in the LLFS nicotine-unexposed sample (p = 0.524). A difference was observed when considering an interaction with nicotine exposure for the LLFS sample (nicotine-exposed vs. nicotine-unexposed; p = 0.097). Beyond rs113288603, we observed evidence for possible associations of rs56113850 * C and rs12461964 * G (the other two alleles identified by NMR GWAS^8^) with increased hearing loss symptoms in nicotine-exposed subjects (p = 0.053 and p = 0.089, respectively; Supplemental Table 3).

**Table 1:** Association between *CYP2A6* rs113288603 and hearing loss symptoms in discovery and replication cohorts (WHI and LLFS, respectively).

Cohort	Nicotine Status	N	Allele_Effect_	Allele Frequency	Beta	SE	P	Z_Interaction_	P_Interaction_
WHI	Exposed	3401	T	0.10	−0.018	0.004	5.75 × 10^−5^	−2.62	8.67 × 10^−3^
	Unexposed	3245			−0.001	0.005	0.8708		
LLFS	Exposed	2077		0.09	−0.249	0.151	0.098	−1.66	0.097
	Unexposed	2548			0.085	0.133	0.524

Considering smoking behaviors, *CYP2A6* rs113288603 also showed a nominal association with smoking status (current vs. former smokers): carriers of the rs113288603 * T (protective for hearing loss) allele are less likely to be current smokers than non-carriers (p = 0.047). We also observed another nominal association between the *CYP2A6* * *V110L* (rs72549435) and years of smoking: carriers of *CYP2A6* * *L110* allele smoke more years than non-carriers (p = 0.015). No other associations were observed between *CYP2A6* alleles and smoking behaviors (Supplemental Table 3).

**Figure 2:**
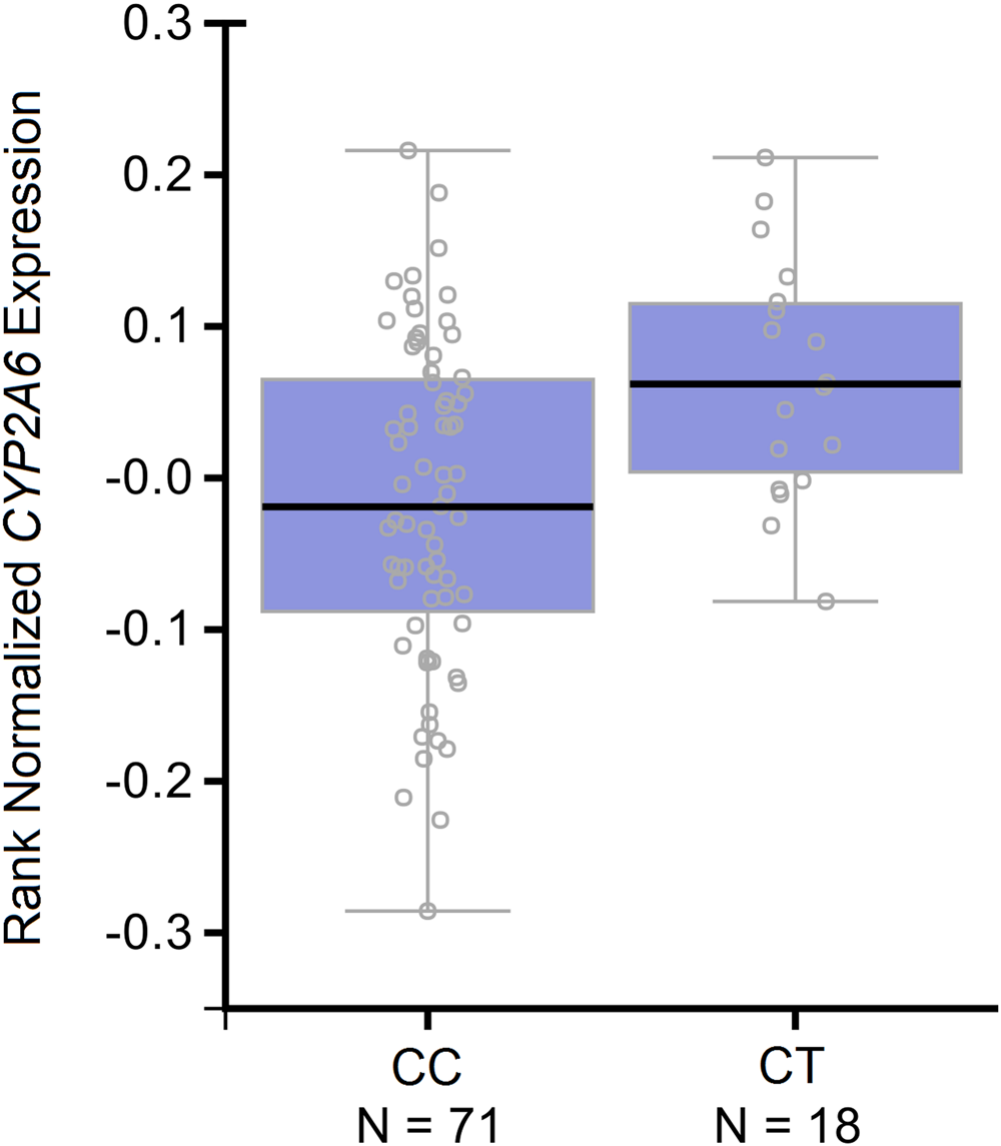
*CYP2A6* expression across rs113288603 genotypes in cerebellar hemisphere.

To attain a biological understanding of how rs113288603 affects *CYP2A6* function, we investigated its effects on *CYP2A6* mRNA expression across human tissues using data from the GTEx consortium (Supplemental Table 4). We observed that rs113288603 * T (protective with respect to hearing loss) is significantly associated with increased *CYP2A6* expression in cerebellar hemisphere (p = 9.9 × 10^−4^; Fig. 2). Comparing rs113288603-expression association with those of rs56113850 * C and rs12461964 * G (the other two alleles identified by NMR GWAS^8^), we observed that, differently from rs113288603, rs56113850 * C and rs12461964 * G are associated with increased *CYP2A6* expression in peripheral tissues (rs56113850: liver p = 1.4 × 10^−3^, lung p = 5.2 × 10^−4^, and ovary p = 5.4 × 10^−5^; rs12461964: liver p = 1.5 × 10^−5^; Supplemental Table 4).

## Discussion

Age-related hearing loss is a common condition among older individuals that contributes to substantially reduced quality of life^31^. In the United States, approximately 33% of the population between ages of 65 and 74 are subject to hearing loss, and after 75 years of age about 50% of the population have hearing problems (data from the National Institute on Deafness and Other Communication Disorders available at https://www.nidcd.nih.gov/health/age-related-hearing-loss). There are many known causes of age-related hearing loss, including age-induced changes to the structure of the inner and middle ear, and also complex changes along the nerve pathways from the ear to the brain^32^. Epidemiological studies, such as The Epidemiology of Hearing Loss Study^17^ and National Health and Nutrition Examination Survey 1999–2004^18^, have shown that the major risk factors for age-related hearing loss are noise exposure and smoking. While the mechanism for noise-associated hearing loss is reasonably well understood, this is not the case for nicotine exposure. Our current PheWAS for *CYP2A6* alleles identified association with hearing loss symptoms in nicotine-exposed elderly subjects. This result supports a harmful effect of smoking on auditory function mechanistically. Indeed, the *CYP2A6* association with hearing loss highlights how nicotine metabolism is a relevant pathway in this pathological condition. Further information is provided by the allele identified: rs113288603 is associated with *CYP2A6* expression in the brain (specifically in cerebellar hemisphere). In a published NMR GWAS^8^, rs113288603 was the most significant independent signal (p_cond_ = 7.03 × 10^−25^) after conditioning for the overall top SNP rs56113850 (p = 5.77 × 10^−86^). As reported above and also in a previous study^10^, rs56113850 is associated with *CYP2A6* hepatic expression, consistent with its association with urinary and serum NMR (i.e. the minor allele C is associated with increased expression and with increased NMR). A similar situation pertains for *CYP2A6* rs12461964 (i.e., concordant effect directions of hepatic expression and NMR). Conversely, rs113288603 appears to be different from these variants; it is not associated with *CYP2A6* hepatic expression. This variant’s minor allele T correlates positively with *CYP2A6* expression in cerebellar hemisphere and the effect direction is in the opposite direction with respect to serum and urinary NMR^8, 9^, i.e., increased *CYP2A6* expression is associated with lower NMR. However, the association of rs113288603 with *CYP2A6* expression in cerebellar hemisphere is in line with its protective effect on hearing loss symptoms (i.e., increased gene expression potentially associated with increased rate of nicotine metabolism in this tissue relevant to the auditory function). This indicates that the *CYP2A6* gene may present different tissue-specific regulatory mechanisms. Rs56113850 and rs12461964 have strong effects on *CYP2A6* hepatic regulation with consistent consequences on urinary and serum NMR; rs113288603 has a limited effect on hepatic regulation with reduced contribution on urinary and serum NMR (rs113288603 signal was genome-wide significant after adjusting the analysis for rs56113850). Conversely, this same variant, rs113288603, has a strong effect on *CYP2A6* brain regulation (particularly in cerebellar hemisphere) and this is protective with respect to hearing loss symptoms. Rs56113850 and rs12461964 seem to provide a minimal contribution to *CYP2A6* brain regulation because they appear to be protective with respect to hearing loss symptoms with effect directions opposite with respect to their associations with hepatic expression and serum and urine NMR. Effect directions of the *CYP2A6* allele associations with NMR, gene expression, and age-related hearing loss symptoms are summarized in Table 2.

**Table 2:** Effect directions of the *CYP2A6* allele associations with NMR, gene expression, and age-related hearing loss symptoms.

Phenotype	rs113288603 * T	rs56113850 * C	rs12461964 * G	Source
serum and urinary NMR	↓NMR (Genome-wide significance)	↑NMR (Genome-wide significance)	↑NMR (Genome-wide significance)	GWAS (Loukola *et al*.^8^; Patel *et al*.^9^)
*CYP2A6* Expression	↑Cerebellar hemisphere (Tissue-wide significance)	↑Liver (Tissue-wide significance)	↑Liver (Tissue-wide significance)	GTEx
Age-related Hearing Loss	↓Symptoms (Phenome-wide significance)	↑Symptoms (p < 0.1)	↑Symptoms (p < 0.1)	current study

Chronic cigarette smoking has been linked to structural changes in several brain regions, including cerebellum^33^, and *CYP2A6* variation mediates some functional brain changes in smokers^34^. These previous findings are consistent with the conclusion that brain nicotine or nicotine metabolite concentration can shape brain circuits, and raise the possibility that some of the toxicity they cause can be irreversible. Our observation of the association of the rs113288603 * T allele with age-related hearing loss symptoms and *CYP2A6* expression in cerebellar hemisphere is consistent with and validated by these prior findings.

Multiple studies have confirmed the role of cerebellum in auditory function^20, 21^, nicotinic cholinergic receptors are present in this brain region^35^, and hearing loss is one known symptom of cerebellar stroke syndromes^36^. On these bases, our PheWAS results can be explained by the following hypothesis: smokers with rs113288603 * T have increased *CYP2A6* expression in cerebellum that results with consequent altered brain exposure to nicotine and some of its metabolites, and thus protects these subjects from the nicotine-induced changes associated with age-related hearing loss. Specifically, the association of *CYP2A6* rs113288603 with age-related hearing loss observed in nicotine-exposed individuals is mediated by the involvement of *CYP2A6* in nicotine metabolism (not by the limited effect on smoking quantity observed). This, apparently, has the effect of reducing the long term effects of nicotine on brain regions involved in hearing function. The protein product of *CYP2A6* has many other physiological roles, however, our observation ties the effect on hearing directly to exposure to nicotine. This finding contributes to the literature regarding the many toxic effects of nicotine on the human body. Beyond its addictive effects, nicotine can alter important physiological functions, and potentially increase risk for several pathological conditions^13, 14^.

In conclusion, our PheWAS identified novel evidence supporting the role of *CYP2A6* variation as an important mediator of at least one smoking consequence. In particular, our data indicate that genetic variation in the nicotine metabolism pathway can mediate the effect of smoking on age-related hearing loss. Our available sample size was rather limited; we predict, based on the likely link of this variant to the biology of quantitative nicotine exposure, that with better sample sizes, the range of medical consequences of nicotine use associated to this variant will grow. Hearing loss in the elderly population is associated with reduced quality of life and impaired psychological and motor skills that can lead to social isolation and depression^31^. A better understanding of age-related hearing loss may permit us to develop more effective preventive strategies and therapeutic approaches that can improve the quality of life of the growing elderly population. Additionally, knowledge of this genotype could identify smokers who should be evaluated for possible hearing loss, regardless of whether or not they report symptoms; although human subject studies will be required to ascertain whether the effect exerted by this one variant is clinically important. Further, there is the possibility that this finding may lead to identification of drug targets whereby this risk can be addressed pharmacologically, in subjects who are unable to cease smoking. Finally, we conclude based on our current and previous results that the genetic investigations of specific population categories, such as nicotine-exposed subjects, alcoholics, or individuals exposed to combat or trauma, can successfully identify mechanisms that differ from those present in the general population^26, 37–39^.

## Electronic supplementary material

Supplemental Tables
