# Supplementary material for: Phenome-wide association study for CYP2A6 alleles: rs113288603 is associated with hearing loss symptoms in elderly smokers
Source: Sci Rep. 2017 Apr 21;7:1034. doi: 10.1038/s41598-017-01098-4 (PMC5430682; doi:10.1038/s41598-017-01098-4)
Supplement: Supplementary file 1 — Supplemental Tables [file 41598_2017_1098_MOESM1_ESM.pdf]

Phenome-wide association study for *CYP2A6* alleles: rs113288603 is associated with hearing loss symptoms in elderly smokers

Renato Polimanti[1], Kevin P. Jensen[1], and Joel Gelernter[1,2]

[1]Department of Psychiatry, Yale University School of Medicine and VA CT Healthcare Center, West Haven, CT, United States

[2]Departments of Genetics and Neuroscience, Yale University School of Medicine, New Haven, CT, United States

Running Title: *CYP2A6* and age-related hearing loss

Corresponding Author: Renato Polimanti, PhD. Department of Psychiatry, Yale University School of Medicine, VA CT 116A2, 950 Campbell Avenue, West Haven, CT 06516, United States. Tel: +1 (203) 932-5711 x5745. Fax: +1 (203) 937-3897. E-mail: renato.polimanti@yale.edu

Supplemental Table 1: Details about phenotypic traits extracted from WHI dataset.

Dataset name	Variable Label	Original Variable Name(s)	Variable Name (present study)	Category
f42_dbgap_rel1.dat	BMImax-BMImin	combined(BMImax;BMImin)	BMIdiff	Anthropometric traits
f42_dbgap_rel1.dat	Maximum adult weight (pounds)+Height at 18 (feet)	combined(WGTMAX;HGTAG18F)	BMImax	Anthropometric traits
f42_dbgap_rel1.dat	Minimum adult weight (pounds)+Height at 18 (feet)	combined(WGTMIN;HGTAG18F)	BMImin	Anthropometric traits
f80_dbgap_rel1.dat	BMI	BMIX	BMIX	Anthropometric traits
f42_dbgap_rel1.dat	Height at 18 (feet)	HGTAG18F	HGTAG18F	Anthropometric traits
f80_dbgap_rel1.dat	BMI	modified(BMIX)	ObesityIgrade	Anthropometric traits
f80_dbgap_rel1.dat	BMI	modified(BMIX)	ObesityIIgrade	Anthropometric traits
f80_dbgap_rel1.dat	BMI	modified(BMIX)	ObesityIIIgrade	Anthropometric traits
f34_dbgap_rel1.dat	Weight during adult life, lbs	modified(WGTADULT)	WGTADULT_gain	Anthropometric traits
f34_dbgap_rel1.dat	Weight during adult life, lbs	modified(WGTADULT)	WGTADULT_lost	Anthropometric traits
f34_dbgap_rel1.dat	Weight during adult life, lbs	modified(WGTADULT)	WGTADULT_same	Anthropometric traits
f34_dbgap_rel1.dat	Weight during adult life, lbs	modified(WGTADULT)	WGTADULT_updown	Anthropometric traits
f42_dbgap_rel1.dat	Weight at birth	WGTBRTH	WGTBRTH	Anthropometric traits
f42_dbgap_rel1.dat	Max weight (age 18-29)	WGTMAX18	WGTMAX18	Anthropometric traits
f42_dbgap_rel1.dat	Max weight (age 30-39)	WGTMAX30	WGTMAX30	Anthropometric traits

f42_dbgap_rel1.dat	Max weight (age 40-49)	WGTMAX40	WGTMAX40	Anthropometric traits
f42_dbgap_rel1.dat	Max weight (age 50-59)	WGTMAX50	WGTMAX50	Anthropometric traits
f42_dbgap_rel1.dat	Max weight (age 60-69)	WGTMAX60	WGTMAX60	Anthropometric traits
f42_dbgap_rel1.dat	Max weight (age 70+)	WGTMAX70	WGTMAX70	Anthropometric traits
f42_dbgap_rel1.dat	Min weight (age 18-29)	WGTMIN18	WGTMIN18	Anthropometric traits
f42_dbgap_rel1.dat	Min weight (age 30-39)	WGTMIN30	WGTMIN30	Anthropometric traits
f42_dbgap_rel1.dat	Min weight (age 50-59)	WGTMIN50	WGTMIN50	Anthropometric traits
f42_dbgap_rel1.dat	Min weight (age 60-69)	WGTMIN60	WGTMIN60	Anthropometric traits
f80_dbgap_rel1.dat	Waist hip ratio	WHRX	WHRX	Anthropometric traits
f34_dbgap_rel1.dat	Number times weight went up/down >10 lbs	YOYO10LB	YOYO10LB	Anthropometric traits
outc_bc_dbgap_rel4.dat/f2_dbgap_rel1.dat/f30_dbgap_rel1.dat	Breast cancer ever	combined(BRCA_F2;BRCA_F30)	BRCA_PLUS	Cancer
f30_dbgap_rel1.dat	Cancer ever	CANC_F30	CANC_F30	Cancer
outc_bc_dbgap_rel4.dat/f2_dbgap_rel1.dat	Colorectal Cancer	combined(COLORECTAL;COLON_F2)	COLOR_PLUS	Cancer
outc_bc_dbgap_rel4.dat/f2_dbgap_rel1.dat	Endometrial Cancer	combined(ENDMTRL;ENDO_F2)	ENDO_PLUS	Cancer
outc_cancer_dbgap_rel4.dat	Lung Cancer	LUNG	LUNG	Cancer
outc_bc_dbgap_rel4.dat/f2_dbgap_rel1.dat/f30_dbgap_rel1.dat	Skin/Melanoma cancer ever	combined(SKIN;MELANOMA)	SKIN_PLUS	Cancer
f30_dbgap_rel1.dat	Angina ever	ANGINA	ANGINA	Cardiovascular traits
f30_dbgap_rel1.dat	Atrial fibrillation ever	ATRIALFB	ATRIALFB	Cardiovascular traits
f2_dbgap_rel1.dat	Bleeding problem ever	BLDPROB	BLDPROB	Cardiovascular traits
ecg_dbgap_rel1/rel2.dat	Bradycardia	combined(BRADYCAR;BRADY)	BRADY	Cardiovascular traits

f30_dbgap_rel1.dat	Cardiac catheterization ever	CARDCATH	CARDCATH	Cardiovascular traits
f30_dbgap_rel1.dat	Cardiovascular disease ever	CVD	CVD	Cardiovascular traits
f80_dbgap_rel1.dat	Diastolic BP	DIAS	DIAS	Cardiovascular traits
f2_dbgap_rel1.dat	DVT ever	DVT	DVT	Cardiovascular traits
ecg_mi_nova_dbgap_rel1.dat	Incident novacode	ECGINCDNTNOVA	ECGINCDNTNOVA	Cardiovascular traits
f38/f37_dbgap_rel1.dat	Heart racing or skipping beats	HEARTRAC	HEARTRAC	Cardiovascular traits
f30_dbgap_rel1.dat	Hypertension ever	HYPT	HYPT	Cardiovascular traits
f30_dbgap_rel1.dat	Age told of hypertension	HYPTAGE	HYPTAGE	Cardiovascular traits
ecg_dbgap_rel2.dat	LVH by Minnesota Code	LVH_MINN	LVH_MINN	Cardiovascular traits
ecg_dbgap_rel1	Major ventricular conduction defects	MAJVENT	MAJVENT	Cardiovascular traits
ecg_dbgap_rel2.dat	MI by Minnesota Code	MC_MI	MC_MI	Cardiovascular traits
f2_dbgap_rel1.dat	MI (myocardial infarction) ever	MI	MI	Cardiovascular traits
ecg_dbgap_rel1/rel2.dat	Minor Ventricular Conduction Defect/aberration	MINVENT	MINVENT	Cardiovascular traits
ecg_dbgap_rel1/rel2.dat	Minnesota code F1	MINF1	MNF1	Cardiovascular traits
ecg_dbgap_rel1/rel2.dat	Minnesota code F5	MINF5	MNF5	Cardiovascular traits
ecg_dbgap_rel1/rel2.dat	Minnesota code L4	MNL4	MNL4	Cardiovascular traits
ecg_dbgap_rel1/rel2.dat	Minnesota code L5	MNL5	MNL5	Cardiovascular traits
ecg_dbgap_rel1/rel2.dat	Minnesota code V1	MNV1	MNV1	Cardiovascular traits
ecg_dbgap_rel1/rel2.dat	Minnesota code V4	MNV4	MNV4	Cardiovascular traits
ecg_dbgap_rel1/rel2.dat	Minnesota code V5	MNV5	MNV5	Cardiovascular traits
f30_dbgap_rel1.dat	Peripheral arterial disease ever	PAD	PAD	Cardiovascular traits
ecg_dbgap_rel1.dat	Prolonged PR interval	PROPRINT	PROPRINT	Cardiovascular traits

f80_dbgap_rel1.dat	Systolic BP+Diastolic BP	combined(SYST;DIAS)	PulsePressure	Cardiovascular traits
ecg_dbgap_rel1.dat	Short PR interval	SHPRINT	SHPRINT	Cardiovascular traits
f2_dbgap_rel1.dat	Stroke ever	STROKE	STROKE	Cardiovascular traits
f80_dbgap_rel1.dat	Systolic BP	SYST	SYST	Cardiovascular traits
f2_dbgap_rel1.dat	TIA (transient ischemic attack) ever	TIA	TIA	Cardiovascular traits
f34_dbgap_rel1.dat	Drink coffee each day	COFFEE	COFFEE	Diet Habits
f60_item_dbgap_rel1.dat	Coffee or tea (all types), med serv/day	COFFEE	COFFEEQ	Diet Habits
f34_dbgap_rel1.dat	Number of regular cups of coffee, day	CUPREG	CUPREG	Diet Habits
f60_nutr_dbgap_rel1.dat	Dietary Caffeine (mg)	F60CAFF	F60CAFF	Diet Habits
f60_nutr_dbgap_rel1.dat	Dietary Energy (kcal)	F60ENRGY	F60ENRGY	Diet Habits
f60_nutr_dbgap_rel1.dat	Daily Fruit Consumption (med portion)	F60FRUIT	F60FRUIT	Diet Habits
f60_nutr_dbgap_rel1.dat	Daily Vegetable Consumption (med portion)	F60VEG	F60VEG	Diet Habits
f34_dbgap_rel1.dat	Drank 12 alcoholic beverages ever	ALC12DR	ALC12DR	Drinking Behaviors
f42_dbgap_rel1.dat	12 or more drinks of alcohol	ALCOHLOS	ALCOHLOS	Drinking Behaviors
f34_dbgap_rel1.dat	Alcohol intake	ALCOHOL	ALCOHOL	Drinking Behaviors
f60/F35/F34_nutr_dbgap_rel1.dat	Alcohol servings per week	F60ALCWK	ALCWK	Drinking Behaviors
f60_item_dbgap_rel1.dat	Beer, med serv/day	BEER	BEER	Drinking Behaviors
f35_dbgap_rel1.dat	Beer - frequency	BEERFREQ	BEERFREQ	Drinking Behaviors
f35_dbgap_rel1.dat	Beer - serving size	BEERSERV	BEERSERV	Drinking Behaviors
f42_dbgap_rel1.dat	Drinks of alcohol (age 14-17)	DRNKAG14	DRNKAG14	Drinking Behaviors

f42_dbgap_rel1.dat	Drinks of alcohol (age 18-22)	DRNKAG18	DRNKAG18	Drinking Behaviors
f42_dbgap_rel1.dat	Drinks of alcohol (age 23-29)	DRNKAG23	DRNKAG23	Drinking Behaviors
f42_dbgap_rel1.dat	Drinks of alcohol (age 30-49)	DRNKAG30	DRNKAG30	Drinking Behaviors
f42_dbgap_rel1.dat	Drinks of alcohol (age 50)	DRNKAG50	DRNKAG50	Drinking Behaviors
f60_nutr_dbgap_rel1.dat	Dietary Alcohol	F60ALC	ALC	Drinking Behaviors
f35_dbgap_rel1.dat	Liquor - frequency	LIQRFREQ	LIQRFREQ	Drinking Behaviors
f35_dbgap_rel1.dat	Liquor - serving size	LIQRSERV	LIQRSERV	Drinking Behaviors
f60_item_dbgap_rel1.dat	Liquor, med serv/day	LIQUOR	LIQUOR	Drinking Behaviors
f60_item_dbgap_rel1.dat	Wine, med serv/day	WINE	WINE	Drinking Behaviors
f35_dbgap_rel1.dat	Wine - frequency	WINEFREQ	WINEFREQ	Drinking Behaviors
f35_dbgap_rel1.dat	Wine - serving size	WINESERV	WINESERV	Drinking Behaviors
f38/f37_dbgap_rel1.dat	Bloating or gas	BLOATING	BLOATING	Gastrointestinal traits
f38/f37_dbgap_rel1.dat	Constipation	CONSTIP	CONSTIP	Gastrointestinal traits
f30_dbgap_rel1.dat	Diverticulitis ever	DIVERTIC	DIVERTIC	Gastrointestinal traits
f30_dbgap_rel1.dat	Part of intestines removed ever	INTESTRM	INTESTRM	Gastrointestinal traits
f30_dbgap_rel1.dat	Stomach of duodenal ulcer ever	STOMULCR	STOMULCR	Gastrointestinal traits
f38/f37_dbgap_rel1.dat	Upset stomach or belly pain	UPSTOM	UPSTOM	Gastrointestinal traits
f38/f37_dbgap_rel1.dat	General aches or pains	ACHES	ACHES	General Health
f30_dbgap_rel1.dat	Arthritis ever	ARTHRIT	ARTHRIT	General Health
f30_dbgap_rel1.dat	Asthma ever	ASTHMA	ASTHMA	General Health
f38/f37_dbgap_rel1.dat	Have trouble getting back to sleep	BACKSLP	BACKSLP	General Health
f38/f37_dbgap_rel1.dat	How much bodily pain	BODPAIN	BODPAIN	General Health
f30_dbgap_rel1.dat	Cataract ever	CATARACT	CATARACT	General Health

f37_dbgap_rel1.dat	Leak urine when cough, laugh	CGHINCON	CGHINCON	General Health
f2_dbgap_rel1.dat	Other long-term illness	combined(OTHCHRON; f2other)	CHRON	General Health
f38/f37_dbgap_rel1.dat	Clumsiness	CLUMSY	CLUMSY	General Health
f38/f37_dbgap_rel1.dat	Coughing or wheezing	COUGH	COUGH	General Health
f2_dbgap_rel1.dat	Diabetes ever	DIAB	DIAB	General Health
f2_dbgap_rel1.dat	Age first told had diabetes	DIABAGE	DIABAGE	General Health
f38/f37_dbgap_rel1.dat	Diarrhea	DIARRHEA	DIARRHEA	General Health
f38/f37_dbgap_rel1.dat	Dizziness	DIZZY	DIZZY	General Health
f30_dbgap_rel1.dat	Emphysema ever	EMPHYSEM	EMPHYSEM	General Health
f38/f37_dbgap_rel1.dat	Energy/fatigue	ENERFAT	ENERFAT	General Health
f38/f37_dbgap_rel1.dat	Fall asleep during quiet activity	FALLSLP	FALLSLP	General Health
f38/f37_dbgap_rel1.dat	How often leaked urine	FRQINCON	FRQINCON	General Health
f30_dbgap_rel1.dat	High blood calcium	HIBLDCA	GALLBS	General Health
f38/f37_dbgap_rel1.dat	General health construct	GENHLTH	GENHLTH	General Health
f30_dbgap_rel1.dat	Glaucoma ever	GLAUCOMA	GLAUCOMA	General Health
f30_dbgap_rel1.dat	Goiter ever	GOITER	GOITER	General Health
f38/f37_dbgap_rel1.dat	Headaches or migraines	HEADACHE	HEADACHE	General Health
f38/f37_dbgap_rel1.dat	Hearing loss	HEARLOSS	HEARLOSS	General Health
f38/f37_dbgap_rel1.dat	Heartburn	HEARTBRN	HEARTBRN	General Health
f30_dbgap_rel1.dat	Kidney or bladder stones ever	KIDNEYST	HIBLDCA	General Health
f30_dbgap_rel1.dat	High cholesterol requiring pills ever	HICHOLRP	HICHOLRP	General Health
f38/f37_dbgap_rel1.dat	My health is excellent	HLTHEXCL	HLTHEXCL	General Health

f38/f37_dbgap_rel1.dat	I am as healthy as anybody	HLTHYANY	HLTHYANY	General Health
f38/f37_dbgap_rel1.dat	How many hours of sleep	HRSSLP	HRSSLP	General Health
f38/f37_dbgap_rel1.dat	Increase appetite	HUNGRY	HUNGRY	General Health
f37_dbgap_rel1.dat	leak limit activities	INCONLMT	INCONLMT	General Health
f38/f37_dbgap_rel1.dat	Ever leaked urine	INCONT	INCONT	General Health
f38_dbgap_rel1.dat	Joint pain or stiffness	JNTPAIN	JNTPAIN	General Health
f30_dbgap_rel1.dat	Gallbladder disease or gallstones ever	GALLBS	KIDNEYST	General Health
f38/f37_dbgap_rel1.dat	How much urine do you lose	LEAKAMT	LEAKAMT	General Health
f37_dbgap_rel1.dat	Phys/Accomplished less	LESSACCP	LESSACCP	General Health
f37_dbgap_rel1.dat	Phys/limited kind of work	LESSKNDP	LESSKNDP	General Health
f37_dbgap_rel1.dat	Phys/cut down on time spent	LESSWRKP	LESSWRKP	General Health
f2_dbgap_rel1.dat	Liver disease ever	LIVERDIS	LIVERDIS	General Health
f38_dbgap_rel1.dat	Low back pain	LOWBACKP	LOWBACKP	General Health
f38/f37_dbgap_rel1.dat	Did you take medication for sleep	MEDSLEEP	MEDSLEEP	General Health
f30_dbgap_rel1.dat	Migraine headaches ever	MIGRAINE	MIGRAINE	General Health
f37_dbgap_rel1.dat	Muscle tension aches or soreness	MSCLACHE	MSCLACHE	General Health
f30_dbgap_rel1.dat	None of listed medical conditions ever	NACOND	NACOND	General Health
f38/f37_dbgap_rel1.dat	Did you nap during the day	NAP	NAP	General Health
f38/f37_dbgap_rel1.dat	Nausea	NAUSEA	NAUSEA	General Health
f38_dbgap_rel1.dat	Neck pain	NECKPAIN	NECKPAIN	General Health

f38/f37_dbgap_rel1.dat	Night sweats	NIGHTSWT	NIGHTSWT	General Health
f30_dbgap_rel1.dat	Thyroid nodule ever	NODULE	NODULE	General Health
f38/f37_dbgap_rel1.dat	Decreased appetite	NOHUNGER	NOHUNGER	General Health
f37_dbgap_rel1.dat	No longer leak urine	NOINCON	NOINCON	General Health
f30_dbgap_rel1.dat	Osteoporosis ever	OSTEOPOR	OSTEOPOR	General Health
f30_dbgap_rel1.dat	Overactive thyroid ever	OVRTHY	OVRTHY	General Health
f38/f37_dbgap_rel1.dat	Pain construct	PAIN	PAIN	General Health
f37_dbgap_rel1.dat	How much did pain interfere	PAININT	PAININT	General Health
f38/f37_dbgap_rel1.dat	Role limitations due to physical health	PHYLIMIT	PHYLIMIT	General Health
f38/f37_dbgap_rel1.dat	Physical functioning construct	PHYSFUN	PHYSFUN	General Health
f38/f37_dbgap_rel1.dat	Typical night's sleep	QUALSLP	QUALSLP	General Health
f38/f37_dbgap_rel1.dat	Restless and fidgety	RESTLESS	RESTLESS	General Health
f30_dbgap_rel1.dat	Rheumatoid arthritis ever	RHEUMAT	RHEUMAT	General Health
f38/f37_dbgap_rel1.dat	I get sick easier than others	SICKEASY	SICKEASY	General Health
f38/f37_dbgap_rel1.dat	Skin dryness or scaling	SKINDRY	SKINDRY	General Health
f38/f37_dbgap_rel1.dat	Sleep disturbance construct	SLPDSTRB	SLPDSTRB	General Health
f37_dbgap_rel1.dat	Leak when I am sleeping	SLPINCON	SLPINCON	General Health
f38/f37_dbgap_rel1.dat	Did you snore	SNORE	SNORE	General Health
f37_dbgap_rel1.dat	Trouble falling asleep or staying asleep	STAYSLP	STAYSLP	General Health
f38/f37_dbgap_rel1.dat	Swelling of hands or feet	SWELLHND	SWELLHND	General Health
f38/f37_dbgap_rel1.dat	Symptom construct	SYMPTOM	SYMPTOM	General Health

f30_dbgap_rel1.dat	Thyroid gland problem ever	THYROID	THYROID	General Health
f37_dbgap_rel1.dat	Getting tired very easily	TIREEASY	TIREEASY	General Health
f37_dbgap_rel1.dat	Leak when can't get to toilet	TOINCON	TOINCON	General Health
f38/f37_dbgap_rel1.dat	Trouble with vision	TRBSEE	TRBSEE	General Health
f38/f37_dbgap_rel1.dat	Did you have trouble falling asleep	TRBSLEEP	TRBSLEEP	General Health
f38/f37_dbgap_rel1.dat	Tremors	TREMORS	TREMORS	General Health
f30_dbgap_rel1.dat	Underactive thyroid ever	UNDTHY	UNDTHY	General Health
f38/f37_dbgap_rel1.dat	Did you wake up earlier than planned	UPEARLY	UPEARLY	General Health
f38/f37_dbgap_rel1.dat	Did you wake up several times	WAKENGHT	WAKENGHT	General Health
f37_dbgap_rel1.dat	Phys/difficulty perform work	WRKDIFFP	WRKDIFFP	General Health
f38/f37_dbgap_rel1.dat	Activities of Daily Living Construct	ACTDLY	ACTDLY	Physical Activity
f34_dbgap_rel1.dat	Times per week of very hard exercise	modified(HRDEX)	HRDEX_Bin	Physical Activity
f34_dbgap_rel1.dat	Times per week of very hard exercise	modified(HRDEX)	HRDEX_quan	Physical Activity
f34_dbgap_rel1.dat	Very hard exercise 3 times/wk at age 18	HRDEX18	HRDEX18	Physical Activity
f34_dbgap_rel1.dat	Very hard exercise 3 times/wk at age 35	HRDEX35	HRDEX35	Physical Activity
f34_dbgap_rel1.dat	Very hard exercise 3 times/wk at age 50	HRDEX50	HRDEX50	Physical Activity
f35_dbgap_rel1.dat	Episodes moderate to strenuous activity >=20 min/week	LMSEPI	LMSEPI	Physical Activity

f35_dbgap_rel1.dat	Energy expenditure from mild exercise (MET-hours/week)	MILDEXP	MILDEXP	Physical Activity
f38/f37_dbgap_rel1.dat	Moderate activites	MODACT	MODACT	Physical Activity
f35_dbgap_rel1.dat	Energy expend from moderate exercise (MET-hours/week)	MODEXP	MODEXP	Physical Activity
f35_dbgap_rel1.dat	Strenuous activity episodes per week	SEPIWK	SEPIWK	Physical Activity
f42_dbgap_rel1.dat	Number of hours spent sitting	SITTING	SITTING	Physical Activity
f42_dbgap_rel1.dat	Number of hours spent sleeping	SLEEPING	SLEEPING	Physical Activity
f38/f37_dbgap_rel1.dat	Vigorous activities	VIGACT	VIGACT	Physical Activity
f37_dbgap_rel1.dat	Ambivalence over Emotional Expressivenes	AMBEMOT	AMBEMOT	Psychological traits
f37_dbgap_rel1.dat	Becoming easily annoyed or irritable	ANNOYED	ANNOYED	Psychological traits
f37_dbgap_rel1.dat	Feeling nervous, anxious, on edge	ANXIOUS	ANXIOUS	Psychological traits
f37_dbgap_rel1.dat	Fear others will not approve if negative	APPRVNEG	APPRVNEG	Psychological traits
f37_dbgap_rel1.dat	Think people make bad luck for sympathy	BADLUCK	BADLUCK	Psychological traits
f37_dbgap_rel1.dat	People guilty of bad sexual behavior	BADSEX	BADSEX	Psychological traits
f37_dbgap_rel1.dat	After anger bothered for a long time	BOTHER	BOTHER	Psychological traits
f38/f37_dbgap_rel1.dat	Felt calm and peaceful	CALM	CALM	Psychological traits
f38/f37_dbgap_rel1.dat	Have major conflict with children	modified(CHILCON)	CHILCON	Psychological traits

f38/f37_dbgap_rel1.dat	Have major conflict with children	modified(CHILCON)	CHILCON_FEEL	Psychological traits
f37_dbgap_rel1.dat	Attend clubs/lodges/groups last month	CLUB	CLUB	Psychological traits
f37_dbgap_rel1.dat	Number of people who try to coerce	COERCE	COERCE	Psychological traits
f38/f37_dbgap_rel1.dat	Difficulty concentrating	CONCEN	CONCEN	Psychological traits
f37_dbgap_rel1.dat	Rarely count on good things happening	COUNTGD	COUNTGD	Psychological traits
f38/f37_dbgap_rel1.dat	You had crying spells	CRYSPELL	CRYSPELL	Psychological traits
f37_dbgap_rel1.dat	Express disappointment	DISAPPNT	DISAPPNT	Psychological traits
f38/f37_dbgap_rel1.dat	Felt down in the dumps	DWNDUMPS	DWNDUMPS	Psychological traits
f38/f37_dbgap_rel1.dat	Role limitation due to emotional problem	EMOLIMIT	EMOLIMIT	Psychological traits
f38/f37_dbgap_rel1.dat	Emotional well-being	EMOWELL	EMOWELL	Psychological traits
f38/f37_dbgap_rel1.dat	Did you have a lot of energy	ENERGY	ENERGY	Psychological traits
f38/f37_dbgap_rel1.dat	You enjoyed life	ENJLIF	ENJLIF	Psychological traits
f37_dbgap_rel1.dat	Number of people who exclude you	EXCLUDE	EXCLUDE	Psychological traits
f37_dbgap_rel1.dat	Usually expect the best	EXPCTBST	EXPCTBST	Psychological traits
f37_dbgap_rel1.dat	Experts often no better than I	EXPERTS	EXPERTS	Psychological traits
f38/f37_dbgap_rel1.dat	Felt downhearted and blue	FELTBLUE	FELTBLUE	Psychological traits
f38/f37_dbgap_rel1.dat	You felt depressed	FELTDEP	FELTDEP	Psychological traits
f38/f37_dbgap_rel1.dat	You felt sad	FELTSAD	FELTSAD	Psychological traits
f38/f37_dbgap_rel1.dat	Forgetfulness	FORGET	FORGET	Psychological traits

f38/f37_dbgap_rel1.dat	Did a close friend die	FRIENDIE	FRIENDIE	Psychological traits
f38/f37_dbgap_rel1.dat	Close friend/family have a divorce	FRNDIV	FRNDIV	Psychological traits
f37_dbgap_rel1.dat	Make friends because friends are useful	FRNDSUSE	FRNDSUSE	Psychological traits
f38/f37_dbgap_rel1.dat	You, family, friend lose job or retire	FRNJOB	FRNJOB	Psychological traits
f38/f37_dbgap_rel1.dat	Did you feel full of pep	FULLPEP	FULLPEP	Psychological traits
f37_dbgap_rel1.dat	Someone to do something fun with	FUN	FUN	Psychological traits
f38/f37_dbgap_rel1.dat	In general, health is	GENHEL	GENHEL	Psychological traits
f37_dbgap_rel1.dat	Someone to give good advice	GOODADVC	GOODADVC	Psychological traits
f37_dbgap_rel1.dat	Someone to have a good time with	GOODTIME	GOODTIME	Psychological traits
f38/f37_dbgap_rel1.dat	Have you been happy	HAPPY	HAPPY	Psychological traits
f37_dbgap_rel1.dat	Someone to help understand a problem	HLPPROB	HLPPROB	Psychological traits
f38/f37_dbgap_rel1.dat	I expect my health to get worse	HLTHWORS	HLTHWORS	Psychological traits
f37_dbgap_rel1.dat	Most people are honest due to fear	HONEST	HONEST	Psychological traits
f37_dbgap_rel1.dat	Always hopeful about future	HOPEFUL	HOPEFUL	Psychological traits
f37_dbgap_rel1.dat	Hostility Construct	HOSTIL	HOSTIL	Psychological traits
f42_dbgap_rel1.dat	Hours/day spent sitting or lying	INACT	INACT	Psychological traits
f37_dbgap_rel1.dat	How much does leakage bother	INCONDIS	INCONDIS	Psychological traits
f38/f37_dbgap_rel1.dat	Currently married or intimate	modified(MARRIED)	INTIMATE	Psychological traits

f38/f37_dbgap_rel1.dat	Extent phys or emotional probs interfere	INTSOC	INTSOC	Psychological traits
f38/f37_dbgap_rel1.dat	Time physical/emotional probs interfere	INTSOC2	INTSOC2	Psychological traits
f37_dbgap_rel1.dat	Usually people around know when angry	KNWANGRY	KNWANGRY	Psychological traits
f38/f37_dbgap_rel1.dat	Emotional/Accomplished less	LESSACCE	LESSACCE	Psychological traits
f38_dbgap_rel1.dat	Emotional/Worked less carefully	LESSCARE	LESSCARE	Psychological traits
f38/f37_dbgap_rel1.dat	Emotional/Cut down on time spent	LESSWRKE	LESSWRKE	Psychological traits
f38/f37_dbgap_rel1.dat	Life event construct #1 (0,1 scoring)	LFEVENT1	LFEVENT1	Psychological traits
f38/f37_dbgap_rel1.dat	Life event construct #2 (0-3 scoring)	LFEVENT2	LFEVENT2	Psychological traits
f37_dbgap_rel1.dat	Most people would lie to get ahead	LIE	LIE	Psychological traits
f38/f37_dbgap_rel1.dat	Rate quality of life	LIFEQUAL	LIFEQUAL	Psychological traits
f37_dbgap_rel1.dat	Someone to listen when need to talk	LISTEN	LISTEN	Psychological traits
f37_dbgap_rel1.dat	Someone to love you/make you feel wanted	LOVE	LOVE	Psychological traits
f38/f37_dbgap_rel1.dat	Have a major accident or disaster	modified(MAJACC)	MAJACC_FEEL	Psychological traits
f38/f37_dbgap_rel1.dat	Have major problems with money	modified(MONPROB)	MONPROB_FEEL	Psychological traits
f38/f37_dbgap_rel1.dat	Mood swings	MOODSWNG	MOODSWNG	Psychological traits
f37_dbgap_rel1.dat	Expect more good things than bad	MOREGOOD	MOREGOOD	Psychological traits

f37_dbgap_rel1.dat	Negative Emotional Expressiveness (NEE)	NEGEMOT	NEGEMOT	Psychological traits
f37_dbgap_rel1.dat	Number of people who get on nerves	NERVES	NERVES	Psychological traits
f38/f37_dbgap_rel1.dat	Have you been a very nervous person	NERVOUS	NERVOUS	Psychological traits
f37_dbgap_rel1.dat	No one cares what happens to you	NOCARE	NOCARE	Psychological traits
f37_dbgap_rel1.dat	Trouble concentrating on things, reading	NOCONCEN	NOCONCEN	Psychological traits
f37_dbgap_rel1.dat	People inwardly don't like to help	NOHELP	NOHELP	Psychological traits
f37_dbgap_rel1.dat	Hardly ever expect things to go my way	NOTMYWAY	NOTMYWAY	Psychological traits
f37_dbgap_rel1.dat	Optimism Construct	OPTIMISM	OPTIMISM	Psychological traits
f37_dbgap_rel1.dat	Take orders from someone who knew less	ORDERS	ORDERS	Psychological traits
f37_dbgap_rel1.dat	Having an anxiety attack -- feel fear or panic	PANIC	PANIC	Psychological traits
f38/f37_dbgap_rel1.dat	You felt people disliked you	PEOPDIS	PEOPDIS	Psychological traits
f37_dbgap_rel1.dat	Have a pet	PET	PET	Psychological traits
f38/f37_dbgap_rel1.dat	Did a pet die	PETDIE	PETDIE	Psychological traits
f38/f37_dbgap_rel1.dat	Shortened CES-D/DIS screening instrument	PSHTDEP	PSHTDEP	Psychological traits
f37_dbgap_rel1.dat	Times attend religious service/church	RELGTIME	RELGTIME	Psychological traits
f37_dbgap_rel1.dat	Religion gives strength and comfort	RELSTRN	RELSTRN	Psychological traits
f37_dbgap_rel1.dat	People demand more respect than give	RESPECT	RESPECT	Psychological traits

f37_dbgap_rel1.dat	Feeling restless so hard to sit still	RESTLSIT	RESTLSIT	Psychological traits
f38/f37_dbgap_rel1.dat	Your sleep was restless	RESTSLP	RESTSLP	Psychological traits
f38/f37_dbgap_rel1.dat	Felt sad for two weeks or more	SAD2WK	SAD2WK	Psychological traits
f38/f37_dbgap_rel1.dat	Felt sad for two or more years	SAD2YRS	SAD2YRS	Psychological traits
f38_dbgap_rel1.dat	Felt sad much of past year	SADMUCH	SADMUCH	Psychological traits
f38/f37_dbgap_rel1.dat	Satisfied with sex frequency	SATFRQSX	SATFRQSX	Psychological traits
f38/f37_dbgap_rel1.dat	How satisfied with quality of life	SATLIFE	SATLIFE	Psychological traits
f38/f37_dbgap_rel1.dat	How satisfied sexually	SATSEX	SATSEX	Psychological traits
f37_dbgap_rel1.dat	If angered, cause scene in public place	SCENEPUB	SCENEPUB	Psychological traits
f38/f37_dbgap_rel1.dat	Sexual activity in last year	SEXACTIV	SEXACTIV	Psychological traits
f38/f37_dbgap_rel1.dat	Worried sex activity will affect healh	SEXWORRY	SEXWORRY	Psychological traits
f37_dbgap_rel1.dat	Someone to share private worries/fears	SHARE	SHARE	Psychological traits
f38/f37_dbgap_rel1.dat	Social functioning	SOCFUNC	SOCFUNC	Psychological traits
f37_dbgap_rel1.dat	Social Strain Construct	SOCSTRN	SOCSTRN	Psychological traits
f37_dbgap_rel1.dat	Social Support Construct	SOCSUPP	SOCSUPP	Psychological traits
f38/f37_dbgap_rel1.dat	Did your spouse have a serious illness	SPOUSILL	SPOUSILL	Psychological traits
f37_dbgap_rel1.dat	Usually suppress anger	SUPPRESS	SUPPRESS	Psychological traits
f38/f37_dbgap_rel1.dat	Did you feel tired	TIRED	TIRED	Psychological traits
f38/f37_dbgap_rel1.dat	Feeling tired	TIRED2	TIRED2	Psychological traits

f37_dbgap_rel1.dat	Number of people who ask too much	TOOMUCH	TOOMUCH	Psychological traits
f37_dbgap_rel1.dat	Safer to trust nobody	TRUSTNO	TRUSTNO	Psychological traits
f37_dbgap_rel1.dat	Argue to convince people of truth	TRUTH	TRUTH	Psychological traits
f37_dbgap_rel1.dat	Most people are unfair to gain profit	UNFAIR	UNFAIR	Psychological traits
f37_dbgap_rel1.dat	Rate current sense of well-being	WELBEING	WELBEING	Psychological traits
f38/f37_dbgap_rel1.dat	Did you feel worn out	WORNOUT	WORNOUT	Psychological traits
f37_dbgap_rel1.dat	Expect something that can will go wrong	WRONG	WRONG	Psychological traits
f32_dbgap_rel1.dat	Age at First Birth	AGEFBIR	AGEFBIR	Reproductive traits
f32_dbgap_rel1.dat	Age at last bleeding	ANYMENSA	ANYMENSA	Reproductive traits
f32_dbgap_rel1.dat	Breast Disease	BRSTDIS	BRSTDIS	Reproductive traits
f32_dbgap_rel1.dat	How old when first breastfed	BRSTFDAF	BRSTFDAF	Reproductive traits
f32_dbgap_rel1.dat	How old when last breastfed	BRSTFDAL	BRSTFDAL	Reproductive traits
f32_dbgap_rel1.dat	How many months total	BRSTFDM	BRSTFDM	Reproductive traits
f32_dbgap_rel1.dat	Number of months breastfed	BRSTFDMO	BRSTFDMO	Reproductive traits
f32_dbgap_rel1.dat	Breastfeed at least one month	BRSTFEED	BRSTFEED	Reproductive traits
f32_dbgap_rel1.dat	How many live births	BRTHLIVN	BRTHLIVN	Reproductive traits
f32_dbgap_rel1.dat	Ever still births	BRTHSTLN	BRTHSTLN	Reproductive traits
f20_dbgap_rel1.dat	Cervical dysplasia ever	CERVDYS	CERVDYS	Reproductive traits
f32_dbgap_rel1.dat	Ever tubal pregnancies	ECTPREG	ECTPREG	Reproductive traits
f32_dbgap_rel1.dat	Full term pregnancy ever	FULLTRMR	FULLTRMR	Reproductive traits

f32_dbgap_rel1.dat	Number of Pregnancies	GRAVID	GRAVID	Reproductive traits
f38/f37_dbgap_rel1.dat	Hot flashes	HOTFLASH	HOTFLASH	Reproductive traits
f2_dbgap_rel1.dat	Hysterectomy ever	HYST	HYST	Reproductive traits
f2_dbgap_rel1.dat	Age at hysterectomy	HYSTAGE	HYSTAGE	Reproductive traits
f32_dbgap_rel1.dat	Age at first period	MENARCHE	MENARCHE	Reproductive traits
f32_dbgap_rel1.dat	Age at menopause	MENO	MENO	Reproductive traits
f32_dbgap_rel1.dat	Age at last regular period	MENOPSEA	MENOPSEA	Reproductive traits
f32_dbgap_rel1.dat	Age at first hot flash	MENPSYAF	MENPSYAF	Reproductive traits
f32_dbgap_rel1.dat	Hot flashes or night sweats	MENPSYMP	MENPSYMP	Reproductive traits
f32_dbgap_rel1.dat	Were periods regular	MENSREG	MENSREG	Reproductive traits
f32_dbgap_rel1.dat	Age at first regular period	MENSREGA	MENSREGA	Reproductive traits
f32_dbgap_rel1.dat	One year without period	MENSWO1Y	MENSWO1Y	Reproductive traits
f32_dbgap_rel1.dat	How many miscarriages or unspecified type of pregnancies	MISCARYUNSPEC	MISCARYUNSPEC	Reproductive traits
f32_dbgap_rel1.dat	Tried becoming pregnant >1 yr	NOCNCEIV	NOCNCEIV	Reproductive traits
f32_dbgap_rel1.dat	Number of Live Births	NUMLIVER	NUMLIVER	Reproductive traits
f32_dbgap_rel1.dat	One or both ovaries removed	OOPH	OOPH	Reproductive traits
f32_dbgap_rel1.dat	Age when ovaries removed	OOPHA	OOPHA	Reproductive traits
f32_dbgap_rel1.dat	Number of Term Pregnancies	PARITY	PARITY	Reproductive traits
f32_dbgap_rel1.dat	Ever been pregnant	PREG	PREG	Reproductive traits
f32_dbgap_rel1.dat	Ever have full-term pregnancy	PREG6M	PREG6M	Reproductive traits

f32_dbgap_rel1.dat	Age at first term pregnancy	PREG6MAF	PREG6MAF	Reproductive traits
f32_dbgap_rel1.dat	Age at last term pregnancy	PREG6MAL	PREG6MAL	Reproductive traits
f32_dbgap_rel1.dat	How many times term pregnancy	PREG6MN	PREG6MN	Reproductive traits
f32_dbgap_rel1.dat	How many times pregnant	PREGNUM	PREGNUM	Reproductive traits
f32_dbgap_rel1.dat	Ever had tubes tied	TUBTIED	TUBTIED	Reproductive traits
f32_dbgap_rel1.dat	Age when tubes tied	TUBTIEDA	TUBTIEDA	Reproductive traits
f38/f37_dbgap_rel1.dat	Pain or burning while urinating	URINPAIN	URINPAIN	Reproductive traits
f38/f37_dbgap_rel1.dat	Vaginal or genital discharge	VAGDIS	VAGDIS	Reproductive traits
f38/f37_dbgap_rel1.dat	Vaginal or genital dryness	VAGDRY	VAGDRY	Reproductive traits
f38/f37_dbgap_rel1.dat	Vaginal or genital irritation	VAGITCH	VAGITCH	Reproductive traits
f42_dbgap_rel1.dat	Lived with smoker after age 18	ADLVSMK	ADLVSMK	Smoking Behaviors
f42_dbgap_rel1.dat	Lived with smoker as a child	CHLVSMK	CHLVSMK	Smoking Behaviors
f35_dbgap_rel1.dat	How many cigarettes per day	CIGSDAY	CIGSDAY	Smoking Behaviors
f34_dbgap_rel1.dat	Age quit smoking regularly	QSMOKAGE	QSMOKAGE	Smoking Behaviors
f34_dbgap_rel1.dat	Quit smoking because of health problems	QSMOKHP	QSMOKHP	Smoking Behaviors
f34_dbgap_rel1.dat	Age started smoking cigarettes regularly	SMOKAGE	SMOKAGE	Smoking Behaviors
f34_dbgap_rel1.dat	Smoking status	SMOKING	SMOKING	Smoking Behaviors
f34_dbgap_rel1.dat	Smoked to lose weight	SMOKWGT	SMOKWGT	Smoking Behaviors

f34_dbgap_rel1.dat	Years as regular smoker	SMOKYRS	SMOKYRS	Smoking Behaviors
f20_dbgap_rel1.dat	Current Health Care Provider	CAREPROV	CAREPROV	Social Status
f20_dbgap_rel1.dat	Marital status	modifed(MARITAL)	DIVORCED	Social Status
f20_dbgap_rel1.dat	Highest grade finished in school	EDUC	EDUC	Social Status
f20_dbgap_rel1.dat	Total family income (before taxes)	INCOME	INCOME	Social Status
f42_dbgap_rel1.dat	Job 1/2/3 - Age started	combined(JOBAGE1/2/3)	JOBAGE	Social Status
f42_dbgap_rel1.dat	Job 1/2/3 - Years worked	combined(JOBY1/2/3)	JOBY	Social Status
f37_dbgap_rel1.dat	Live alone	LIVALN	LIVALN	Social Status
f37_dbgap_rel1.dat	Living Alone	LIVALOR	LIVALOR	Social Status
f38/f37_dbgap_rel1.dat	Have a major accident or disaster	MAJACC	MAJACC	Social Status
f20_dbgap_rel1.dat	Marital status	modified(MARITAL)	MARRIED	Social Status
f38/f37_dbgap_rel1.dat	Have major problems with money	MONPROB	MONPROB	Social Status
f20_dbgap_rel1.dat	Marital status	MARITAL	NEVERMARRIED	Social Status
f20_dbgap_rel1.dat	No insurance	NOINS	NOINS	Social Status
f20_dbgap_rel1.dat	Partner highest level of education	PEDUC	PEDUC	Social Status

Supplemental Table 2: *CYP2A6* variants investigated in the present study. Imputation details (info score and allele frequency) are referred to the WHI cohort.

rsID	Variant	Position (chr19)	Imputed Allele	Allele Frequency	Imputation Info score
rs12461964	intron (NMR GWAS)	41341229	G	0.3302	0.7239
rs28399463	N418D	41350587	T	0.9756	0.7763
rs28399454	V365M	41351267	T	0.0817	0.7804
rs56113850	intron (NMR GWAS)	41353107	C	0.4714	0.721
rs56256500	R203C	41354171	A	0.0103	0.5489
rs28399440	F118L	41354660	A	0.9865	0.589
rs72549435	V110L	41355738	C	0.9867	0.6896
rs28399433	-48T>G	41356379	A	0.8883	0.7416
rs113288603	intron (NMR GWAS)	41362293	T	0.09	0.8202

Supplemental Table 3: *CYP2A6* results related to hearing loss and smoking behaviors. Due to the low allele frequency, it was not possible some variants with respect to certain phenotypes.

Phenotype Id	rsID	Allele	Beta	P
HEARLOSS	rs113288603	T	-0.0177	5.75E-05
	rs56113850	C	0.0055	0.05328
	rs12461964	G	0.0055	0.08938
	rs28399463	T	-0.01	0.2639
	rs28399454	T	0.0039	0.4421
	rs28399433	A	-0.0034	0.4645
	rs72549435	C	0.0062	0.6528
CIGSDAY	rs28399463	T	-0.0453	0.2414
	rs12461964	G	-0.0092	0.5099
	rs56113850	C	-0.0067	0.5831
	rs28399433	A	-0.0085	0.6689
	rs28399454	T	0.0094	0.6691
	rs72549435	C	0.0174	0.7735
	rs113288603	T	-0.0026	0.89
QSMOKAGE	rs28399433	A	-0.1489	0.221
	rs56113850	C	-0.0916	0.2272
	rs113288603	T	0.1031	0.3965
	rs28399454	T	-0.0753	0.5858
	rs28399463	T	0.1009	0.6687
	rs12461964	G	-0.0258	0.7628
	rs72549435	C	-0.0318	0.926
QSMOKHP	rs113288603	T	0.16432	0.2782
	rs28399463	T	0.30778	0.3608
	rs72549435	C	-0.26136	0.5231
	rs56113850	C	0.05308	0.5912
	rs12461964	G	0.03335	0.7658

	rs28399433	A	0.025473	0.8748
	rs28399454	T	0.0004	0.9982
SMOKAGE	rs28399433	A	-0.0052	0.4416
	rs113288603	T	0.0036	0.5778
	rs28399463	T	-0.0061	0.6435
	rs56113850	C	-0.0017	0.6889
	rs12461964	G	0.0013	0.7861
	rs28399454	T	0.002	0.7936
	rs72549435	C	0.0042	0.8342
SMOKING	rs113288603	T	0.0082	0.04787
	rs28399454	T	0.0068	0.1603
	rs72549435	C	0.0151	0.2459
	rs28399433	A	0.0045	0.302
	rs28399463	T	0.0056	0.509
	rs12461964	G	0.0006	0.8429
	rs56113850	C	0.0001	0.9788
SMOKWGT	rs28399454	T	0.24569	0.07018
	rs113288603	T	0.16059	0.1821
	rs72549435	C	0.43068	0.3385
	rs28399433	A	0.12027	0.369
	rs28399463	T	0.20734	0.4443
	rs12461964	G	-0.04248	0.6401
	rs56113850	C	-0.00797	0.9212
SMOKYRS	rs72549435	C	0.5527	0.01507
	rs56113850	C	0.043	0.3447
	rs28399454	T	0.0769	0.3462
	rs12461964	G	0.041	0.4271
	rs28399433	A	-0.0366	0.622
	rs28399463	T	-0.0313	0.8262
	rs113288603	T	-0.0106	0.8806

Supplemental Table 4: rs113288603*T, rs56113850*C, and rs12461964*G associations with *CYP2A6* expression across human tissues (data from GTEx available at http://www.gtexportal.org/).

Tissue	rs113288603*T		rs56113850*C		rs12461964*G	
	Beta	*P*	*Beta*	*P*	*Beta*	*P*
Brain - Caudate (basal ganglia)	0.11	0.66	0.1	0.5	0.12	0.44
Brain - Cerebellar Hemisphere	0.87	0.00099	-0.13	0.43	-0.085	0.61
Brain - Cerebellum	0.52	0.071	-0.2	0.19	-0.1	0.5
Brain - Cortex	0.51	0.067	-0.012	0.94	-0.21	0.21
Brain - Frontal Cortex (BA9)	-0.16	0.58	0.031	0.84	0.12	0.43
Brain - Hippocampus	0.021	0.93	-0.2	0.28	-0.12	0.51
Brain - Hypothalamus	0.057	0.82	0.13	0.44	0.18	0.25
Brain - Nucleus accumbens (basal ganglia)	-0.096	0.71	0.04	0.77	0.055	0.69
Brain - Putamen (basal ganglia)	-0.39	0.16	-0.12	0.39	-0.033	0.84
Breast - Mammary Tissue	0.16	0.35	0.26	0.0077	0.26	0.0077
Cells - Transformed fibroblasts	0.045	0.68	-0.11	0.12	-0.093	0.16
Colon - Sigmoid	0.27	0.19	-0.015	0.9	-0.071	0.53
Esophagus - Gastroesophageal Junction	0.21	0.35	-0.041	0.73	-0.012	0.92
Esophagus - Mucosa	-0.11	0.49	0.081	0.38	0.074	0.44
Esophagus - Muscularis	0.13	0.35	-0.029	0.74	0.067	0.44
Heart - Atrial Appendage	0.25	0.25	0.056	0.62	0.12	0.3
Heart - Left Ventricle	-0.061	0.74	0.31	0.0023	0.23	0.033
Liver	-0.093	0.54	0.26	0.0014	0.36	0.000015
Lung	-0.093	0.44	0.22	0.00052	0.18	0.0075
Muscle - Skeletal	0.0072	0.95	-0.048	0.47	-0.079	0.24
Nerve - Tibial	0.0073	0.95	-0.0017	0.98	0.033	0.68
Ovary	0.033	0.91	0.6	0.000054	0.21	0.24
Pancreas	-0.0032	0.99	-0.15	0.27	-0.15	0.25
Pituitary	0.18	0.51	-0.071	0.63	-0.11	0.44

Prostate	0.2	0.44	0.15	0.41	0.093	0.62
Skin - Not Sun Exposed (Suprapubic)	0.11	0.57	0.29	0.0078	0.28	0.011
Skin - Sun Exposed (Lower leg)	-0.09	0.51	-0.087	0.25	0.045	0.55
Small Intestine - Terminal Ileum	0.31	0.34	-0.019	0.91	0.057	0.72
Spleen	0.032	0.89	-0.087	0.56	0.19	0.21
Stomach	-0.11	0.54	0.078	0.44	0.043	0.68
Testis	0.35	0.074	0.22	0.036	0.28	0.0083
Thyroid	0.25	0.062	-0.13	0.066	-0.15	0.029
Uterus	-0.58	0.06	0.39	0.012	0.39	0.044
Vagina	-1.1	0.0021	0.39	0.025	0.3	0.09
Whole Blood	0.028	0.83	-0.023	0.75	0.045	0.53
